# Meiotic deviations and endoreplication lead to diploid oocytes in female hybrids between bighead catfish (*Clarias macrocephalus*) and North African catfish (*Clarias gariepinus*)

**DOI:** 10.3389/fcell.2024.1465335

**Published:** 2024-08-23

**Authors:** Dmitrij Dedukh, Artem Lisachov, Thitipong Panthum, Worapong Singchat, Yoichi Matsuda, Yukiko Imai, Karel Janko, Kornsorn Srikulnath

**Affiliations:** ^1^ Laboratory of Non-Mendelian Evolution, Institute of Animal Physiology and Genetics, Czech Academy of Sciences, Liběchov, Czechia; ^2^ Animal Genomics and Bioresource Research Unit (AGB Research Unit), Faculty of Science, Kasetsart University, Bangkok, Thailand; ^3^ Institute of Cytology and Genetics, Russian Academy of Sciences, Siberian Branch, Novosibirsk, Russia; ^4^ Department of Gene Function and Phenomics, National Institute of Genetics, Mishima, Japan; ^5^ Department of Biology and Ecology, Faculty of Natural Sciences, University of Ostrava, Ostrava, Czechia; ^6^ Biodiversity Center Kasetsart University (BDCKU), Bangkok, Thailand

**Keywords:** synaptonemal complex, lampbrush chromosomes, telomeric sequence, satellite DNA, clariid catfish

## Abstract

**Introduction:**

Reproductive isolation and hybrid sterility are mechanisms that maintain the genetic integrity of species and prevent the introgression of heterospecific genes. However, crosses of closely related species can lead to complex evolution, such as the formation of all-female lineages that reproduce clonally. Bighead catfish (*Clarias macrocephalus*) and North African catfish (*C. gariepinus*) diverged 40 million years ago. They are cultivated and hybridized in Thailand for human consumption. Male hybrids are sterile due to genome-wide chromosome asynapsis during meiosis. Although female hybrids are sometimes fertile, their chromosome configuration during meiosis has not yet been studied.

**Methods:**

We analyzed meiosis in the hybrid female catfish at pachytene (synaptonemal complexes) and diplotene (lampbrush chromosomes), using immunostaining to detect chromosome pairing and double-stranded break formation, and FISH with species-specific satellite DNAs to distinguish the parental chromosomes.

**Results:**

More than 95% of oocytes exhibited chromosome asynapsis in female hybrid catfish; however, they were able to progress to the diplotene stage and form mature eggs. The remaining oocytes underwent premeiotic endoreplication, followed by synapsis and crossing over between sister chromosomes, similar to known clonal lineages in fish and reptiles.

**Discussion:**

The occurrence of clonal reproduction in female hybrid catfish suggests a unique model for studying gametogenic alterations caused by hybridization and their potential for asexual reproduction. Our results further support the view that clonal reproduction in certain hybrid animals relies on intrinsic mechanisms of sexually reproducing parental species, given their multiple independent origins with the same mechanism.

## 1 Introduction

Reproductive isolation is defined as a mechanism to prevent the production of an offspring between different species. However, various forms of incomplete reproductive isolation in interspecific crosses have been observed in related species (Avise, 2008; Lukhtanov et al., 2020; Caeiro-Dias et al., 2023). Interspecific hybridization, once considered a rare evolutionary phenomenon, is now known to be common in nature and is recognized as a driver of various evolutionary consequences (Abbott et al., 2013). These consequences include the reinforcement of reproductive isolation or the facilitation of genetic exchange between hybridizing species, which leads to the creation of novel genetic combinations and the emergence of new species (Matute et al., 2010; Abbott et al., 2013; Hopkins, 2013; Dufresnes et al., 2016). Hybrid fertility is often affected by genome incompatibility in parental species crosses, which leads to sterility due to deficiency in meiotic chromosome paring and recombination, which disrupts meiosis and leads to formation of aneuploid gametes. However, one intriguing and lesser-known outcome of interspecific hybridization is the formation of fertile hybrids that can reproduce asexually, despite the chromosomal incompatibilities between parental species (Schön et al., 2009; Stöck et al., 2021). In a way, asexual reproduction may represent a remedy for hybrid sterility, and recent studies have indicated a mechanistic link between the two phenomena. These findings suggest that hybrid asexual reproduction emerges as a special type of postzygotic reproductive incompatibility when divergence between parental species increases along the speciation continuum, from freely admixing populations towards isolated species (Janko et al., 2018; Dedukh et al., 2020; Stöck et al., 2021; Marta et al., 2023). At certain intermediate levels of genetic and chromosomal divergence, hybrids may no longer produce reduced recombined gametes but instead exhibit a special type of gametogenic aberration leading to asexuality or clonality (Stöck et al., 2021; Marta et al., 2023).

Naturally occurring asexual hybrids exhibit a broad spectrum of cytological characteristics that produce clonal or hemiclonal gametes. These mechanisms include deviations from canonical gametogenesis and fertilization processes, such as parthenogenesis (the development of an egg without interaction with sperm, occurring in some fishes and reptiles), gynogenesis (the development of an egg after activation by a sperm, but without accepting its genetic material, which is found in various fishes), kleptogenesis (a process in amphibians where both clonally and sexually inherited genomes exist, but excessive genomes are removed after fertilization), and hybridogenesis (a process in various fishes and amphibians where clonally and sexually inherited genomes exist, but excessive genomes are removed during gametogenesis) (Schön et al., 2009; Stöck et al., 2021; Dedukh and Krasikova, 2022). Two mechanisms for unreduced egg formation are generally known in clonal vertebrates. First, in various clonal vertebrates, chromosomes are duplicated prior to meiosis, with each chromosome set paired with its copy, thus avoiding heterospecific synapsis at meiotic prophase (Stenberg and Saura, 2009; Neaves and Baumann, 2011). Premeiotic endoreplication has been found in various hybrid clonal organisms, including spined loaches and dojo loaches (*Cobitis* and *Misgurnus*, Cypriniformes), American salamanders (Ambystomatidae, Caudata), and whiptail lizards (*Aspidoscelis*, Teiidae), mourning geckos (*Lepidodactylus*, Gekkonidae), and Caucasian rock lizards (*Darevskia*, Lacertidae) (Macgregor and Uzzell, 1964; Itono et al., 2006; Lutes et al., 2010; Mogie, 2013; Dedukh et al., 2020; Dedukh et al., 2022a; Dedukh et al., 2024; Spangenberg et al., 2024). After the completion of meiosis I and II, the oocytes regain the original ploidy level of the maternal cells. Second, in mollies (*Poecilia formosa*, Cyprinodontiformes, Teleostei) and gibel and crucian carps (*Carassius gibelio, C. langsdorfii*, Cypriniformes*,* Teleostei), chromosomes are not duplicated and do not synapse or undergo recombination, and they exist only as univalents (Yamashita et al., 1993; Yang et al., 1999; Dedukh et al., 2022b; Lu et al., 2022). Meiosis I (reductional division) is omitted to generate diploid eggs in these species, which allows sister chromatids to divide during meiosis II (equational division) (Monaco et al., 1984; Yamashita et al., 1993; Lu et al., 2022). While generally considered rare in nature, the switch to asexual gametogenesis may actually be a common outcome of interspecific hybridization in some groups of organisms. This phenomenon is regularly manifested as early as the F_1_ generation of hybrids (Marta et al., 2023). Laboratory crosses between related species have demonstrated that the transition to asexual breeding can occur in the F_1_ generation in a sex-specific manner. This suggests that asexual hybrids exploit pre-existing programs in their parental species (Kim and Lee, 1990; Shimizu et al., 2000; Dedukh et al., 2021; Dedukh et al., 2024; Marta et al., 2023). Thus, F_1_ hybrids obtained from related bisexual species are considered good models for elucidating the mechanism of clonal reproduction. This challenges the view that the transition from sexual to asexual reproduction in hybrid vertebrates is rare and that the gametogenic machinery, which forms reduced and recombined gametes, is stable and conserved. Understanding the onset mechanisms of asexual emergence may allow us to create stable asexual lineages in the desired species, which opens new possibilities for aquaculture.

Asian and African lineages of clariid catfish (*Clarias*, Clariidae, Siluriformes) diverged over 40 million years ago, yet several species are still able to hybridize (Pouyaud et al., 2009). In Southeast Asia, aquaculture practices have resulted in the artificial production and farming of hybrids between female bighead catfish (*Clarias macrocephalus*) and male North African catfish (*Clarias gariepinus*) for human consumption. These hybrids combine the good meat quality of bighead catfish with the hardiness, high growth rate, and disease resistance of North African catfish and are widely cultivated in Thailand for meat production (Na-Nakorn et al., 2004; Lisachov et al., 2023). The chromosome numbers differ between North African catfish (2n = 56, with 38 bi-armed and 18 acrocentric chromosomes) and bighead catfish (2n = 54, with 38 bi-armed and 16 acrocentric chromosomes) (Lisachov et al., 2024). Consequently, reproductive failure of their F_1_ hybrids limits the production of the next-generation of individuals. Male hybrids are sterile; not all specimens produce sperm, and any sperm produced is dysfunctional. This sterility is caused by genome-wide synapsis failure during meiotic prophase, which leads to the absence or incomplete pairing of homeologous chromosomes (Ponjarat et al., 2019; Lisachov et al., 2024). By contrast, female hybrids are more likely to be fertile and produce backcross progenies with high fertility (75.5%–87.4%) and low embryo mortality (Na-Nakorn et al., 2004; Abol-Munafi et al., 2006). However, their meiosis has not yet been studied, and their mode of reproduction remains unknown. It has been hypothesized that asexual reproduction occurs during gametogenesis in female hybrid catfish. In this study, the presence or absence of chromosome duplication and synapsis in female gametogenesis was investigated by examining the meiotic chromosome configurations at pachytene and diplotene stages in female hybrids. To identify species-specific chromosomes, species-specific satellite DNA (satDNA) probes, which we developed previously (Lisachov et al., 2024), were used for fluorescence *in situ* hybridization (FISH). Possible mechanisms of unreduced oocyte formation in female hybrid catfish were discussed.

## 2 Material and methods

### 2.1 Specimen collection

Female hybrid catfish were obtained from the fresh food markets in Kamphaeng Saen (Nakhon Pathom Province) and Bangkok in Thailand. The sex of each individual was determined based on external and internal gonadal morphology (Kitano et al., 2007; Ponjarat et al., 2019). The hybrid and its parental species, bighead catfish (*C. macrocephalus*) and North African catfish (*C. gariepinus*), were identified using morphological characteristics and differences in chromosome numbers (Maneechot et al., 2016). Each fish was sacrificed by severing the spinal cord anterior to the dorsal fin and then dissecting the ovary tissue. Pachytene chromosomes were obtained from small juvenile ovaries without visible eggs by observing the synaptonemal complexes (SCs). Diplotene chromosomes were isolated from mature ovaries and egg masses by observing lampbrush chromosomes (LBCs). SCs and LBCs were analyzed in six and seven specimens, respectively. All animal care and experimental procedures were approved by the Animal Experiment Committee of Kasetsart University, Thailand (approval nos. ACKU65-SCI-003, ACKU66-SCI-006, and ACKU66-SCI-014) and concurred with the Regulations on Animal Experiments at Kasetsart University and the ARRIVE guidelines (https://arriveguidelines.org).

### 2.2 SC preparation and immunofluorescent staining

SC preparation was performed according to the method described by Peters et al. (1997) with slight modifications. Hypotonic treatment was omitted, and the ovary pieces were placed directly in a 100 mM sucrose solution. Immunostaining was performed as described by Anderson et al. (1999). The lateral and central elements of the SCs were detected using rabbit polyclonal antibodies against SYCP3 (1:500; ab15093, Abcam, Cambridge, United Kingdom) and chicken polyclonal antibodies against SYCP1 (1:500, home-made). The chicken polyclonal Sycp1 antibody was raised against the N-terminal region (1–408) of the zebrafish Sycp1 protein, as previously reported (Ozaki et al., 2011; Saito et al., 2014). Double-strand break repair loci were detected using chicken polyclonal antibodies against RAD51 recombinase (1:100; GTX00721; GeneTex, Hsinchu, Taiwan). The secondary antibodies used were Cy3-conjugated goat anti-rabbit IgG (1:500; 111-165-144, Jackson ImmunoResearch, West Grove, United States), Alexa-488-conjugated goat anti-rabbit IgG (H + L) (1:200, A-11008, Thermo Fisher Scientific, Waltham, MA, United States), and Alexa-594 goat anti-chicken IgY (H + L) (1:200, A-11042, Thermo Fisher Scientific, Waltham, MA, United States). The antibodies were diluted in PBT buffer [dissolve 0.1% (v/v) Tween 20 in 1 × PBS buffer], which consisted of 0.5% bovine serum albumin and 0.05% Tween-20 in phosphate-buffered saline (1 × PBS). For each slide, 50 μL of the antibody solution was applied to a slide under a coverslip. The slides were incubated with primary antibodies in a humid chamber at room temperature from 3 h to 12 h and washed in PBS with 0.1% Tween-20 three times for 10 min each. They were then incubated with secondary antibodies for 1 h, followed by washing in PBS containing 0.1% Tween-20 three times for 10 min each. After staining, the slides were mounted in Vectashield/DAPI (1.5 mg/mL) anti-fade mounting medium (Vector Laboratories, Burlingame, CA, United States). The fluorescence signals were captured using a Provis AX70 Olympus microscope equipped with a standard fluorescence filter set. Microphotographs of the chromosomes were captured with a CCD camera (DP30W Olympus) using Olympus Acquisition Software and further adjusted using Adobe Photoshop CS6 software.

### 2.3 LBC preparation

LBCs from female hybrid catfish were prepared according to a previous protocol (Gall et al., 1991). After dissection, parts of mature ovaries were placed into OR2 saline [82.5 mM NaCl, 2.5 mM KCl, 1 mM MgCl_2_, 1 mM CaCl_2_, 1 mM Na_2_HPO_4_, 5 mM HEPES (4-(2-hydroxyethyl)-1-piperazineethanesulfonic acid), pH 7.4]. Oocyte nuclei were isolated manually using jeweler forceps (11252, Fine Science Tools GmbH, Heidelberg, Germany) in the isolation medium “5:1” [83 mM KCl, 17 mM NaCl, 6.5 mM Na_2_HPO_4_, 3.5 mM KH_2_PO_4_, 1 mM MgCl_2_, 1 mM DTT (dithiothreitol), pH 7.0–7.2]. The oocyte nuclei were subsequently transferred to the “1:4” medium, a one-fourth strength “5:1” medium supplemented with 0.1% paraformaldehyde and 0.01% 1 M MgCl_2_, to remove the nucleus membrane and release the nucleoplasm into the solution. Nucleoplasm from each oocyte was transferred into glass chambers attached to a slide filled in a “1:4” medium. This method ensured that each chamber contained chromosomes spread from an individual oocyte. The slide was then centrifuged, fixed for 30 min in 2% Paraformaldehyde in 1 × PBS, and post-fixed in 50% ethanol for 5 min and 70% ethanol overnight (at 4°C). Next, the slides were dehydrated in 96% ethanol, air-dried, and either used for FISH, or mounted in Vectashield/DAPI (1.5 mg/mL) (Vector, Burlingame, CA, United States) for direct LBC observation.

### 2.4 FISH procedure

For FISH, two previously developed satDNA probes, CLA-SAT-215 subfamily IV satDNA and CLA-SAT-149 satDNA (Lisachov et al., 2024), were used to identify species-specific chromosomes in the oocytes of female hybrid catfish. The CLA-SAT-215 subfamily IV, specific to *C. gariepinus*, was located in the interstitial region of many chromosome pairs, whereas CLA-SAT-149, specific to *C. macrocephalus*, was located in the q-terminal region of three chromosome pairs. Commercially available biotin-labeled 42-bp oligonucleotides, complementary to (TTAGGG)_n_ sequences specific to the telomeric region, and CLA-SAT-225 satDNA, specific to the pericentromeric region of both *C. macrocephalus* and *C. gariepinus*, were also used to simplify the counting of diplotene chromosomes (Lisachov et al., 2024). Commercially synthesized biotin- or digoxigenin-labeled oligonucleotide probes were used for mapping the repeats on chromosomes (Macrogen Co., Seoul, Korea). Slides with LBCs were denatured separately in 75% formamide in 2 × SSC (saline-sodium citrate buffer; 2 × SSC – 0.3 M NaCl, 30 mM Na_3_C_6_H_5_O_7_) for 3 min at 72°C. The slides were then transferred to ethanol (50%, 70%, and 96%) in ice and air-dried. The probes were diluted in a hybridization mixture including 50% formamide, 10% dextran sulfate, 2 × SSC, 5 ng/μL labeled probe, and 10–50-fold excess of salmon sperm DNA. After the probes were denatured at 86°C for 6 min, they were applied to LBC slides, covered with cover slips, carefully sealed at the edges with rubber cement, and incubated overnight at room temperature in a humid chamber. After hybridization, the slides were washed three times in 0.2 × SSC at 44°C for 5 min each. Biotin-dUTPs and digoxigenin-dUTPs were detected using streptavidin-Alexa Fluor 488 (S11223, Invitrogen, San Diego, CA, United States) and anti-digoxigenin-rhodamine (11207750910, Merck, Darmstadt, Germany), respectively. The slides were washed in 4 × SSC with 0.05% Tween-20, transferred through gradient ethanol (50%, 70%, and 96%), and air-dried. Chromosomal DNA was counterstained with Vectashield/DAPI (1.5 mg/mL) (Vector Laboratories, Burlingame, CA, United States).

### 2.5 Wide-field, fluorescence, and confocal laser scanning microscopy

To assess the presence of germ cells, gonadal tissue fragments were initially analyzed using confocal microscopy. Gonadal tissue fragments were fixed in 2% PFA diluted in 1 × PBS for 4 h and then transferred to 1 × PBS. Prior to analysis, tissue fragments were stained with 0.1% DAPI solution in 1 × PBS, followed by transfer to a drop of DABCO antifade solution on cover slides. A Leica TCS SP5 microscope based on an inverted Leica DMI 6000 CS microscope (Leica Microsystems, Germany) was used for confocal laser scanning microscopy. A diode laser was used to excite DAPI. The specimens were analyzed using the HC PL APO 40× and 63× objectives. Images were captured and processed using LAS AF software (Leica Microsystems, Germany).

### 2.6 Comparison to other types of asexual vertebrates of hybrid origin

To place the type of hybrid reproduction in the context of divergence between parental species, the comparison used by Stöck et al. (2021) and Marta et al. (2023) was followed. Specifically, available sequences of the mitochondrial cytochrome b (*cytb*) gene (OL658606.1 and EU670586.1) and the nuclear Recombination Activation Gene 1 (*RAG1*) (XM_053515093.1 and KJ533271.1) for both parental *Clarias* species were downloaded from GenBank. To estimate their genetic differentiation, comparable to datasets used in previous studies of sexual and asexual hybrids, their K2P corrected nucleotide divergence was calculated. In addition, karyotype data published in Lisachov et al. (2024) were used, and the magnitude of the karyotypic differences was calculated using the Autosomal Karyotype Index (AKD) (Castiglia, 2014). This index is calculated as the sum of the absolute differences in diploid numbers of chromosomes (2n), divided by two, and the absolute differences in the autosomal fundamental numbers of arms (NF), also divided by two.

## 3 Results

**FIGURE 1:**
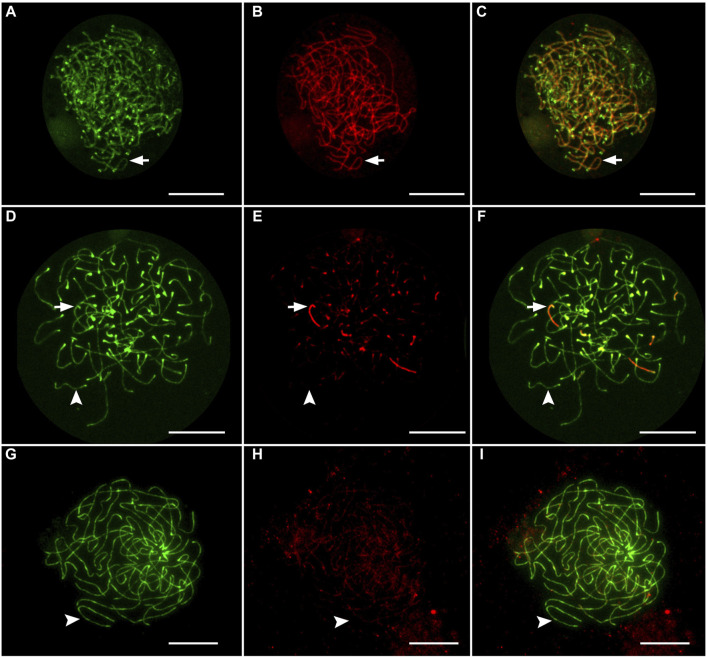
Immunofluorescent staining of lateral element and transverse filaments of synaptonemal complexes in oocytes from female hybrids. Oocytes with exclusively synapsed chromosomes **(A–C)**, with partial synapsis **(D–F)**, and without synapsed chromosomes **(G–I)**. Arrows indicate that synapsed chromosomes showed the presence of both SYCP3-stained (green) lateral elements and SYCP1-stained (red) transverse filaments. Arrowheads indicate that unsynapsed chromosomes that accumulate only lateral elements (SYCP3). Scale bar represents 10 μm.

Gonadal microanatomy of female hybrid catfish showed similar distribution of gonocytes and pachytene clusters between the previtellogenic and vitellogenic oocytes in all individuals (Supplementary Figure S1A, B). In six juvenile hybrid females, meiotic configurations of pachytene chromosomes were analyzed by immunostaining the lateral elements (SYCP3) and the transverse filaments (SYCP1) of the SCs, which enable the distinction between asynapsed and synapsed chromosomes (Blokhina et al., 2019; Dedukh et al., 2020; Dedukh et al., 2022b). The vast majority of pachytene oocytes (n = 360; 96.7%) had fully asynapsed 55 univalent chromosomes in all hybrid individuals used for the pachytene analysis (Clarias_2023_F1, Clarias_2023_F2, Clarias_2024_F1, Clarias_2024_F2, Clarias_2024_F3, Clarias_2024_F4) (Table 1; Figures 1G–I). In some oocytes, occasional synapsis was observed in one to six pairs of chromosomes (Figures 1A–F). However, in the three hybrids (Clarias_2023_F1, Clarias_2023_F2, Clarias_2024_F1), approximately 55 fully synapsed chromosome pairs were detected, indicating that premeiotic genome endoreplication occurred in a small portion of gonial cells (Table 1; Figures 1A–C). RAD51 was absent in most hybrid oocytes, indicating a lack of double-strand break formation (Supplementary Figures S2D–F). However, some pachytene cells were intensively decorated with RAD51, which suggests the presence of double-stranded breaks in some cells (Supplementary Figure S2A–C).

**TABLE 1:** The list of female hybrid catfish examined (in this study) and summary of meiotic chromosome configurations at pachytene and diplotene stages in their oocytes.

Number of individuals	Pachytene		Diplotene	
	Number of oocytes with 55 bivalents	Number of oocytes with 55 univalents	Number of oocytes with 55 bivalents	Number of oocytes with 55 univalents
Clarias_2023_F1	1	27		
Clarias_2023_F2	2	51		
Clarias_2024_F1	9	115		
Clarias_2024_F2	0	124		
Clarias_2024_F3	0	14		
Clarias_2024_F4	0	29		
Clarias_2023_F3			8	7
Clarias_2023_F4			8	8
Clarias_2023_F5			0	18
Clarias_2024_F5			29	0
Clarias_2024_F6			0	27
Clarias_2024_F7			0	21
Clarias_2024_F8			0	16

**FIGURE 2:**
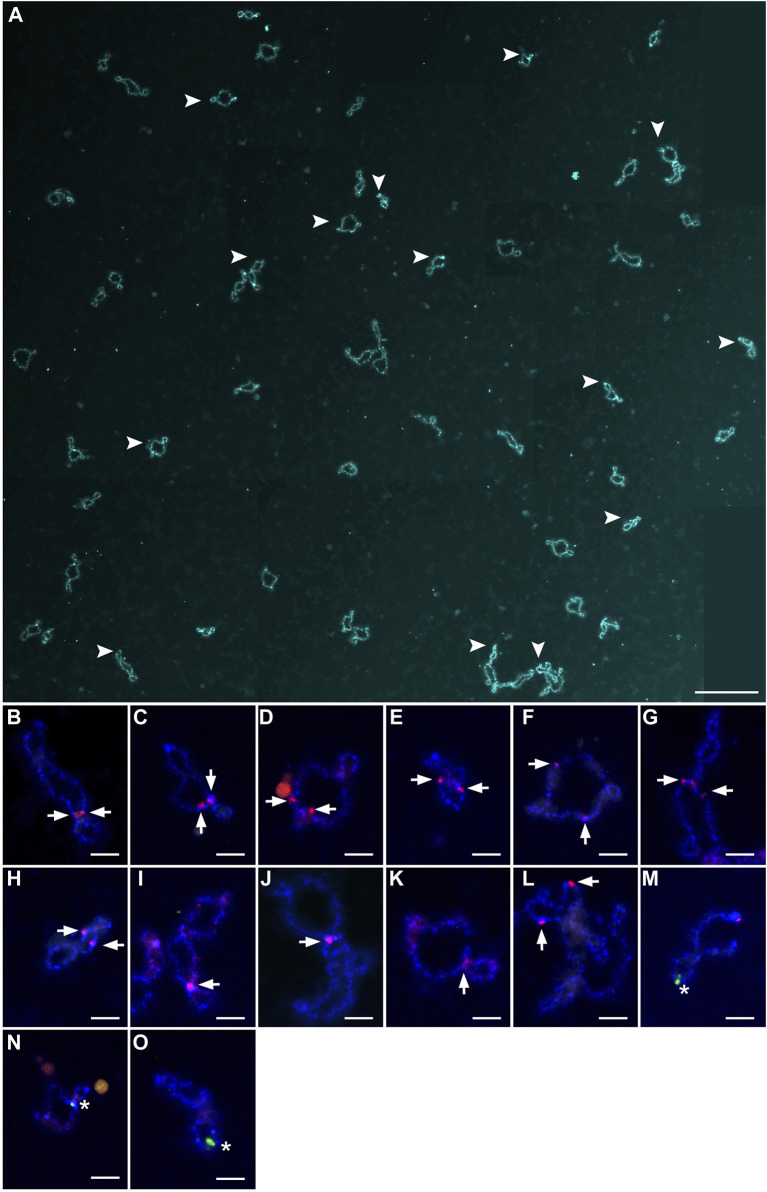
Full lampbrush chromosome set that includes 55 bivalents, which was isolated from individual diplotene oocytes of female hybrid catfish. **(A)** DAPI-stained 55 bivalents of female hybrid catfish. Arrowheads indicate the bivalents that were magnified in **(B–O)**. Scale bar for **(A)** represents 50 μm. Individual bivalents were identified by fluorescence *in situ* hybridization mapping. CLA-SAT-215 satDNA (arrows; red), specific to *Clarias gariepinus* chromosomes, was mapped **(B–L)**, and CLA-SAT-149 satDNA (arrows; green), specific to *Clarias macrocephalus* chromosomes, was mapped **(M–O)**. Scale bars for **(B–O)** represent 5 μm.

**FIGURE 3:**
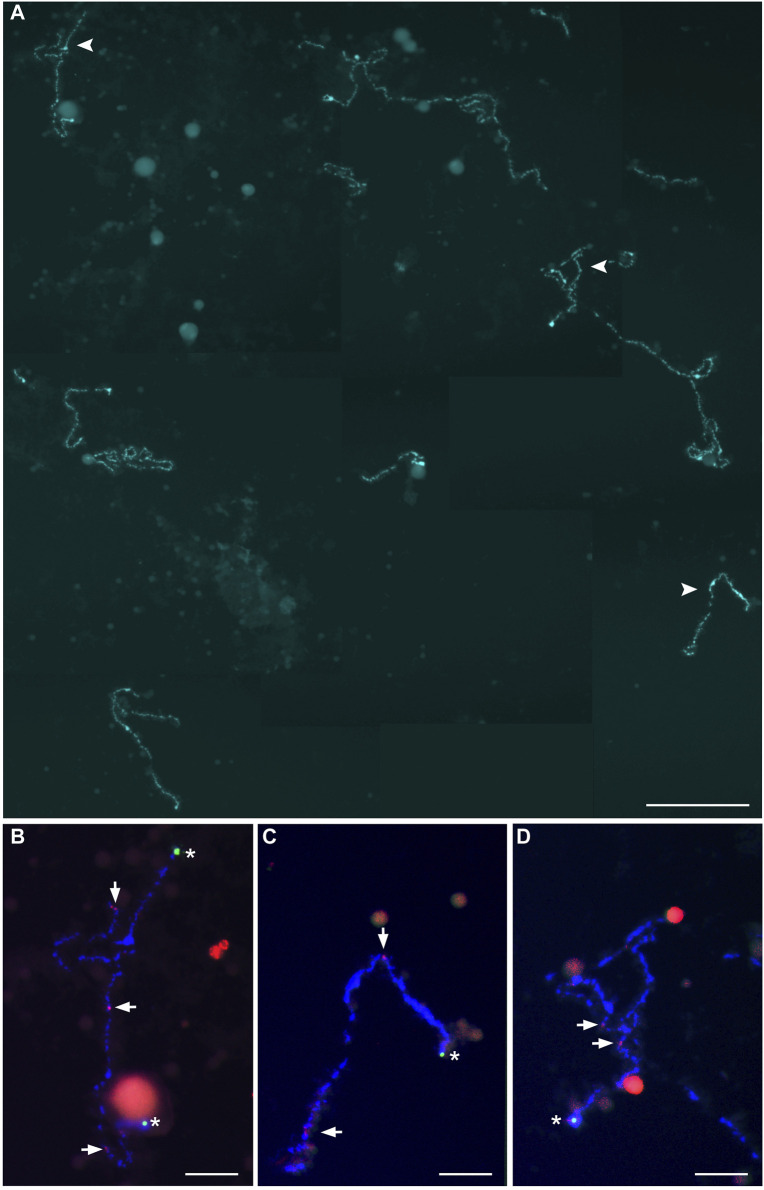
Full lampbrush chromosome set that includes 55 univalents, which was isolated from individual diplotene oocytes of female hybrid catfish. **(A)** DAPI-stained 55 univalents of female hybrid catfish. Arrowheads indicate the bivalents that were magnified in **(B–D)**. Scale bar for **(A)** represents 50 μm. Fluorescence *in situ* hybridization mapping identified CLA-SAT-149 satDNA (asterisks; green), specific to *Clarias macrocephalus* chromosomes, and CLA-SAT-225 satDNA (arrows; red), specific to the pericentromeric region of all chromosomes of both species. Scale bars for **(B–D)** represent 10 μm.

Because homologous chromosome synapsis was not observed in the majority of pachytene oocytes, the oocytes at the diplotene stage were analyzed in another seven hybrid females (Clarias_2023_F3, Clarias_2023_F4, Clarias_2023_F5, Clarias_2024_F5, Clarias_2024_F6, Clarias_2024_F7, Clarias_2024_F8) (Table 1). FISH was applied with telomeric (TTAGGG)_n_ and/or pericentromeric CLA-SAT-225 satDNA probes to simplify chromosomal counting. The chromosomal ends were visualized, which confirmed the formation of univalents at the diplotene stage. In one hybrid female (Clarias_2024_F5), all the oocytes (n = 29) were found with only 55 bivalents (Figure 2; Supplementary Figure S3). FISH with satellite markers revealed signals at similar positions in the interstitial regions on each bivalent chromosome, which suggests that premeiotic endoreplication cause the emergence of homologous chromosome pairs (Figures 2B–O). In four other females (Clarias_2023_F5 and Clarias_2024_F6 to F8), oocytes (n = 18, 27, 21, and 16, respectively) were found with only 55 univalents (Table 1; Figure 3; Supplementary Figures S4, S5). Additionally, in two females (Clarias_2023_F3 and Clarias_2023_F4), oocytes were detected with both 55 bivalents (n = 8 and 8) and 55 univalents (n = 7 and 8) (Table 1). To confirm the presence of genomes of two species in diplotene oocytes, FISH was performed with CLA-SAT-215 satDNA and CLA-SAT-149 satDNA probes in oocytes with bivalents (Figure 2) and with CLA-SAT-149 and pericentromeric CLA-SAT-225 satDNA probes in oocytes with univalents (Figure 3). In oocytes with bivalents, chiasmata were clearly observed between paired chromosomes, which indicates that homologous recombination occurred along each chromosomal pair (Figure 2; Supplementary Figure S3).

Comparison of nucleotide sequences between the two parental taxa indicated K2P values of 0.122 for *cytb* and 0.031 for *RAG1*. Based on the published chromosome characteristics of North African catfish (2n = 56, with 38 bi-armed and 18 acrocentric chromosomes) and bighead catfish (2n = 54, with 38 bi-armed and 16 acrocentric chromosomes), the corresponding AKD value for interspecific differentiation was calculated to be 2.

## 4 Discussion

### 4.1 Diploid female hybrid catfish have two types of oocytes

This study investigated gametogenesis in diploid female hybrids of female bighead catfish (*C. macrocephalus*) and male North African catfish (*C. gariepinus*). Ploidy levels and chromosomal pairing in pachytene and diplotene oocytes were examined by FISH with satDNA markers and telomeric sequences and immunofluorescent staining with SC antibodies. Notably, two types of oocytes were found that differed in ploidy and pairing abilities: 1) diploid oocytes with the original ploidy level and 2) tetraploid oocytes with doubled ploidy due to premeiotic endoreplication. Premeiotic endoreplication allows pairing of the copies of homologous chromosomes during meiosis, which suggests progression through meiosis and the formation of diploid gametes with a genome that is identical to that of the mother. Premeiotic genome endoreplication efficiently resolves issues in homologous chromosome pairing during meiotic prophase and enables clonal reproduction (Lutes et al., 2010; Kuroda et al., 2018; Dedukh et al., 2020; Dedukh et al., 2021; Dedukh et al., 2022a; Marta et al., 2023). Moreover, it appears to be a common trait in hybrid vertebrates that reproduce asexually, as observed in the natural clonal lineages of loaches and other fish, amphibians, and reptiles (Macgregor and Uzzell, 1964; Cuellar, 1971; Stöck et al., 2002; Itono et al., 2006; Lutes et al., 2010; Dedukh et al., 2015; Kuroda et al., 2018; Dedukh et al., 2020; Majtánová et al., 2021; Dedukh et al., 2022a; Dedukh et al., 2024; Spangenberg et al., 2024). However, in all hybrid vertebrates (that reproduce asexually) studied thus far, endoreplication rarely occurs in the germ cells of hybrid individuals (Shimizu et al., 2000; Newton et al., 2016; Dedukh et al., 2021; Dedukh et al., 2022a; Dedukh et al., 2024; Marta et al., 2023). Although tetraploidization of oocytes is rare, oocytes with tetraploid genomes may restore the fertility of some female hybrid catfish, which results in the formation of clonal gametes. While viable progeny have been produced from both tetraploid and diploid oocytes in different hybrid catfish, it cannot be conclusively stated that diploid oocytes are dysfunctional or that females with these oocytes are sterile (Na-Nakorn et al., 2004). Progeny testing by backcross are essential to confirm the asexual reproduction of hybrid catfish females. Additionally, the analysis of ploidy and genome composition of backcrossed progeny will facilitate the comprehension of the role of different oocyte populations in gamete formation.

The ability to undergo genome endoreplication is substantially different between male and female hybrid catfish. Premeiotic genome endoreplication has not been observed in hybrid males (Lisachov et al., 2024), although hybrid females sometimes undergo this process. Although the mechanisms behind sex-specific bias in premeiotic genome endoreplication are unknown, this phenomenon is widespread in nearly all other lineages that reproduce asexually (Itono et al., 2006; Spangenberg et al., 2017; Kuroda et al., 2018; Dedukh et al., 2020; Marta et al., 2023; Dedukh et al., 2024). The sex-specific bias that triggers endoreplication suggests a role for genetic sex determination. However, the transplantation of spermatogonia from male hybrids into females of sexual species within the European loach hybrid complex restored the ability to endoreplicate their gonocyte genomes (Tichopád et al., 2022). This suggests that the initiation of endoreplication, at least in European *Cobitis*, is not directly connected to the genetic sex determination of the individual, but rather to the gonadal environment, which occurs only in the ovary. Alternatively, in *Misgurnus* loaches, endoreplication occurs in hybrids genetically determined to be female hybrids but artificially sex-reverted into males by hormonal treatment (Yoshikawa et al., 2007; Yoshikawa et al., 2009). This suggests that the potential of genome endoreplication in *Misgurnus* loaches does not depend on the phenotypic sex of the individual but is genetically determined.

Most pachytene and diplotene oocytes (88.9%) in diploid female hybrid catfish retained their initial ploidy levels. At the pachytene and diplotene stages of meiosis, the oocytes exhibited full or nearly full asynapsis of the orthologous chromosomes, which existed as univalents. Similar to females, spermatocytes with univalents were exclusively found in diploid male hybrid catfish (Lisachov et al., 2024). Oocytes with aberrant pairing and univalent formation are usually unable to progress beyond pachytene, likely because of their inability to bypass the pachytene checkpoint, which leads to apoptosis (Shimizu et al., 2000; Newton et al., 2016; Dedukh et al., 2021; Dedukh and Krasikova, 2022; Dedukh et al., 2024). However, oocytes with only univalents have reproductive potential in several organisms that reproduce asexually, such as *P. formosa* and *C. gibelio* (Monaco et al., 1984; Yang et al., 1999; Dedukh et al., 2022b; Lu et al., 2022). Similar to *P. formosa* and *C. gibelio*, oocytes with initial ploidy levels lack double-strand break formation and crossover in female hybrid catfish (Dedukh et al., 2022b; Lu et al., 2022). Moreover, an earlier research on achiasmatic meiosis in *P. formosa* revealed downregulation of the gene compared to its sexual parental species, *P. mexicana* (Dedukh et al., 2022b). The Spo11 protein is crucial to form double-strand breaks and initiate recombination at the onset of meiosis (de Massy, 2013; Qu et al., 2021). Similarly, in Spo11 knockout zebrafish females, synapsis and double-strand break formation were prevented, but meiosis still occurred in the oocytes, and progeny were generated. However, these progeny exhibited severe developmental abnormalities, possibly due to egg aneuploidy (Blokhina et al., 2019; Imai et al., 2021). Thus, a similar mechanism for the formation of such oocytes in different groups of organisms may involve the prevention of double-strand breaks and require additional modification of meiotic divisions to produce unreduced gametes. Although the mechanism that underlies the formation of unreduced gametes is not fully understood in *P. formosa* and *C. gibelio*, it has been suggested that reductional division is skipped while equational division occurs correctly, which results in one diploid egg and one polar body (Monaco et al., 1984; Yang et al., 1999; Dedukh et al., 2022b; Lu et al., 2022). In the oocytes of *C. langsdorfii* and *C. gibelio*, a tripolar spindle is formed without univalent attachment during reductional division (Yamashita et al., 1993; Yang et al., 1999). It remains unclear whether oocytes with univalents in female hybrid catfish progress beyond the first meiotic division. However, the presence of occasional pairing and decreased fertility in female hybrids suggests that these oocytes may be unable to complete normal meiotic divisions. Moreover, spermatocytes with only univalents were observed in male hybrid catfish, which showed no chromosome pairing. During meiosis I in these spermatocytes, some chromosomes fail to attach to the spindle and gametogenesis is arrested beyond this stage, which results in decreased fertility and/or complete sterility (Lisachov et al., 2024).

### 4.2 Instant formation of asexual gametogenesis in F_1_ hybrid catfish

Premeiotic genome endoreplication, followed by the formation of diploid clonal gametes, has been observed in F_1_ laboratory crosses of *Cobitis* hybrids, medaka fish (*Oryzias*), and clariid hybrid catfish in this study (Shimizu et al., 2000; Choleva et al., 2012; Dedukh et al., 2022b; Marta et al., 2023). Comparative analyses of parental species by Janko et al. (2018) and Stöck et al. (2021) have suggested that known asexual hybrid vertebrates emerge from crosses of genetically divergent species. Remarkably, the vast majority of asexual hybrids, with the exception of *Darevskia*, frequently emerge from crosses between species with homomorphic sex chromosomes (Stöck et al., 2021). In *Cobitis* hybrids, genetic divergence and chromosomal dissimilarity in sexual species are considered to cause asexual gametogenesis in their hybrids (Marta et al., 2023). North African catfish and bighead catfish diverged more than 40 million years ago, and their diploid numbers differed by one chromosome pair (2n = 56 and 2n = 54, respectively) (Pouyaud et al., 2009; Lisachov et al., 2024). The genetic divergence between both sexual *Clarias* species, measured by *cytb* sequence divergence as a proxy, falls at the higher end of the spectrum of divergences observed in other known cases of fish, amphibian, and reptile species producing asexual hybrids. In these cases, the *cytb* divergence values ranged from approximately 0.05–0.18 (Stöck et al., 2021). To contextualize the observed data regarding sexual species producing hybrids with premeiotic endoreplication, the *RAG1* sequence divergence and AKD measure of karyotype divergence were compared with the data presented by Marta et al. (2023). The obtained values suggest that the genetic divergence between the two parental *Clarias* species (0.031) surpasses the maximum values of interparental divergences known to give rise to hybrids with this type of asexual gametogenesis. These values have so far been reported to range between approximately 0.005 and 0.026, using *RAG1* as a proxy (Marta et al., 2023). By contrast, the karyotypes remained morphologically similar, as indicated by the AKD value of 2, which represents the lower end of divergences (Marta et al., 2023). This suggests that both *Clarias* species are highly genetically differentiated, but their karyotypes have retained greater similarity than other pairs of species known to produce asexual hybrids, especially those using premeiotic endoduplication. However, the exact frequency of chromosomal rearrangements between the two species is unknown, as not all rearrangements may affect the difference of diploid numbers. The alignment of finely assembled genome sequences between two species is required to clarify this issue; however, only the genome of North African catfish has been assembled at the chromosome level (GCF_024256445). Bighead catfish have an XY sex chromosome system (Nguyen et al., 2021a), whereas different lineages of North African catfish are likely to have different sex chromosome systems (Nguyen et al., 2021b; Nguyen et al., 2022; Lisachov et al., 2023). The stock bred in Hungary has an XY sex determination system (Balogh et al., 2023). However, Thai stocks of North African catfish have mixed origins (Chalermwong et al., 2023; Patta et al., 2024) and a polygenic sex determination system (Nguyen et al., 2021b; Nguyen et al., 2022). All sex chromosomes in both catfish species are homomorphic. This suggests that hybrids of North African and bighead catfish exhibit two key characteristics: homomorphic sex chromosomes and substantial genetic divergence. However, the emergence of achiasmatic meiosis in asexual hybrids is not well understood. Different cellular mechanisms are required to prevent chromosome pairing and recombination but allow for progression beyond several meiotic checkpoints. In the *P. formosa*, only a unique hybridization event between the parental species led to such gametogenic alterations and the emergence of stable clonal lineages. Laboratory crosses between the parental species, *P. mexicana* and *P. latipinna*, have failed to create stable asexual clones with gametogenic alterations similar to *P. formosa* (Lampert et al., 2007). In female hybrid catfish, oocytes with initial ploidy levels dominate at the pachytene stage (96.7%). However, in some mature female hybrids studied at the diplotene stage, the proportion of oocytes with an endoreplicated genome is much higher than in younger females studied at the pachytene stage (31.7% on average). This suggests that oocytes with endoreplicated genomes are more likely to progress to the diplotene stage of meiosis.

In the present study, the course of meiosis in female hybrids of North African catfish and bighead catfish was observed for the first time. As these hybrids simultaneously produce two types of oocytes with different ploidy levels and chromosome pairing abilities, they represent a unique model for the study of gametogenic alterations caused by hybridization and the potential for asexual reproduction. Moreover, confirmation of asexual reproduction through further laboratory cross-experiments could make this a valuable technique to improve the production of catfish.

## Data Availability

The datasets presented in this study can be found in online repositories. The names of the repository/repositories and accession number(s) can be found in the article/Supplementary Material.

## References

[B1] Abbott R. Albach D. Ansell S. Arntzen J. W. Baird S. J. Bierne N. (2013). Hybridization and speciation. J. Evol. Biol. 26 (2), 229–246. 10.1111/j.1420-9101.2012.02599.x 23323997

[B2] Abol-Munafi A. B. Liem P. T. Ambak M. A. Siraj S. S. (2006). Effects of maturational hormone treatment on spermatogenesis of hybrid catfish (*Clarias macrocephalus* x *C. gariepinus*). J. Sustain. Sci. Manag. 1 (1), 24–31.

[B3] Anderson L. K. Reeves A. Webb L. M. Ashley T. (1999). Distribution of crossing over on mouse synaptonemal complexes using immunofluorescent localization of MLH1 protein. Genetics 151 (4), 1569–1579. 10.1093/genetics/151.4.1569 10101178 PMC1460565

[B4] Avise I. J. (2008). Clonality: the genetics, ecology, and evolution of sexual abstinence in vertebrate animals. New York: Oxford University Press.

[B5] Balogh R. E. Csorbai B. Guti C. Keszte S. Urbányi B. Orbán L. (2023). Validation of a male-specific DNA marker confirms XX/XY-type sex determination in several Hungarian strains of African catfish (*Clarias gariepinus*). Theriogenology 205, 106–113. 10.1016/j.theriogenology.2023.04.017 37116410

[B6] Blokhina Y. P. Nguyen A. D. Draper B. W. Burgess S. M. (2019). The telomere bouquet is a hub where meiotic double-strand breaks, synapsis, and stable homolog juxtaposition are coordinated in the zebrafish, *Danio rerio* . PLoS Genet. 15 (1), e1007730. 10.1371/journal.pgen.1007730 30653507 PMC6336226

[B7] Caeiro‐Dias G. Brelsford A. Meneses‐Ribeiro M. Crochet P. A. Pinho C. (2023). Hybridization in late stages of speciation: strong but incomplete genome‐wide reproductive isolation and “large Z‐effect” in a moving hybrid zone. Mol. Ecol. 32 (15), 4362–4380. 10.1111/mec.17035 37316984

[B8] Castiglia R. (2014). Sympatric sister species in rodents are more chromosomally differentiated than allopatric ones: implications for the role of chromosomal rearrangements in speciation. Mammal. Rev. 44 (1), 1–4. 10.1111/mam.12009

[B9] Chalermwong P. Panthum T. Wattanadilokcahtkun P. Ariyaraphong N. Thong T. Srikampa P. (2023). Overcoming taxonomic challenges in DNA barcoding for improvement of identification and preservation of clariid catfish species. Genomics Inf. 21 (3), e39. 10.5808/gi.23038 PMC10584641 37813635

[B10] Choleva L. Janko K. De Gelas K. Bohlen J. Šlechtová V. Rábová M. (2012). Synthesis of clonality and polyploidy in vertebrate animals by hybridization between two sexual species. Evolution 66 (7), 2191–2203. 10.1111/j.1558-5646.2012.01589.x 22759295

[B11] Cuellar O. (1971). Reproduction and the mechanism of meiotic restitution in the parthenogenetic lizard *Cnemidophorus uniparens* . J. Morphol. 133 (2), 139–165. 10.1002/jmor.1051330203 5542237

[B12] Dedukh D. Altmanová M. Klíma J. Kratochvíl L. (2022a). Premeiotic endoreplication is essential for obligate parthenogenesis in geckos. Development 149 (7), dev200345. 10.1242/dev.200345 35388415

[B13] Dedukh D. Da Cruz I. Kneitz S. Marta A. Ormanns J. Tichopád T. (2022b). Achiasmatic meiosis in the unisexual Amazon molly, *Poecilia formosa* . Chromosome Res. 30 (4), 443–457. 10.1007/s10577-022-09708-2 36459298 PMC9771850

[B14] Dedukh D. Krasikova A. (2022). Delete and survive: strategies of programmed genetic material elimination in eukaryotes. Biol. Rev. 97 (1), 195–216. 10.1111/brv.12796 34542224 PMC9292451

[B15] Dedukh D. Litvinchuk S. Rosanov J. Mazepa G. Saifitdinova A. Shabanov D. (2015). Optional endoreplication and selective elimination of parental genomes during oogenesis in diploid and triploid hybrid European water frogs. PLoS One 10 (4), e0123304. 10.1371/journal.pone.0123304 25894314 PMC4403867

[B16] Dedukh D. Majtánová Z. Marta A. Pšenička M. Kotusz J. Klíma J. (2020). Parthenogenesis as a solution to hybrid sterility: the mechanistic basis of meiotic distortions in clonal and sterile hybrids. Genetics 215 (4), 975–987. 10.1534/genetics.119.302988 32518062 PMC7404241

[B17] Dedukh D. Marta A. Janko K. (2021). Challenges and costs of asexuality: variation in premeiotic genome duplication in gynogenetic hybrids from *Cobitis taenia* complex. Int. J. Mol. Sci. 22 (22), 12117. 10.3390/ijms222212117 34830012 PMC8622741

[B18] Dedukh D. Altmanová M. Petrosyan R. Arakelyan M. Galoyan E. Kratochvíl L. (2024). Premeiotic endoreplication is the mechanism of obligate parthenogenesis in rock lizards of the genus Darevskia. bioRxiv. 10.1101/2024.02.27.582286 PMC11407861 39288813

[B19] de Massy B. (2013). Initiation of meiotic recombination: how and where? Conservation and specificities among eukaryotes. Annu. Rev. Genet. 47 (1), 563–599. 10.1146/annurev-genet-110711-155423 24050176

[B20] Dufresnes C. Majtyka T. Baird S. J. Gerchen J. F. Borzée A. Savary R. (2016). Empirical evidence for large X-effects in animals with undifferentiated sex chromosomes. Sci. Rep. 6 (1), 21029. 10.1038/srep21029 26868373 PMC4751523

[B21] Gall J. G. Murphy C. Callan H. G. Wu Z. (1991). Lampbrush chromosomes. Met. Cell Biol. 36, 149–166. 1811131

[B22] Hopkins R. (2013). Reinforcement in plants. New Phytol. 197 (4), 1095–1103. 10.1111/nph.12119 23495388

[B23] Imai Y. Olaya I. Sakai N. Burgess S. M. (2021). Meiotic chromosome dynamics in zebrafish. Front. Cell Dev. Biol. 9, 757445. 10.3389/fcell.2021.757445 34692709 PMC8531508

[B24] Itono M. Morishima K. Fujimoto T. Bando E. Yamaha E. Arai K. (2006). Premeiotic endomitosis produces diploid eggs in the natural clone loach, *Misgurnus anguillicaudatus* (Teleostei: cobitidae). J. Exp. Zool. A Comp. Exp. Biol. 305 (6), 513–523. 10.1002/jez.a.283 16526047

[B25] Janko K. Pačes J. Wilkinson‐Herbots H. Costa R. J. Roslein J. Drozd P. (2018). Hybrid asexuality as a primary postzygotic barrier between nascent species: on the interconnection between asexuality, hybridization and speciation. Mol. Ecol. 27 (1), 248–263. 10.1111/mec.14377 28987005 PMC6849617

[B26] Kim I. Lee J. (1990). Diploid-triploid complex of the spined loach *Cobitis sinensis* and *C. longicorpus* (Pisces, Cobitidae). Korean J. Ichthyol. 2, 203–210.

[B27] Kitano J. Mori S. Peichel C. L. (2007). Sexual dimorphism in the external morphology of the threespine stickleback (*Gasterosteus aculeatus*). Copeia 2007 (2), 336–349. 10.1643/0045-8511(2007)7[336:SDITEM]2.0.CO;2

[B28] Kuroda M. Fujimoto T. Murakami M. Yamaha E. Arai K. (2018). Clonal reproduction assured by sister chromosome pairing in dojo loach, a teleost fish. Chromosome Res. 26 (4), 243–253. 10.1007/s10577-018-9581-4 29882067

[B29] Lampert K. P. Steinlein C. Schmid M. Fischer P. Schartl M. (2007). A haploid-diploid-triploid mosaic of the Amazon molly, *Poecilia formosa* . Cytogenet. Genome Res. 119 (1–2), 131–134. 10.1159/000109629 18160792

[B30] Lisachov A. Nguyen D. H. Panthum T. Ahmad S. F. Singchat W. Ponjarat J. (2023). Emerging importance of bighead catfish (*Clarias macrocephalus*) and North African catfish (*C. gariepinus*) as a bioresource and their genomic perspective. Aquaculture 573, 739585. 10.1016/j.aquaculture.2023.739585

[B31] Lisachov A. Panthum T. Dedukh D. Singchat W. Ahmad S. F. Wattanadilokcahtkun P. (2024). Genome-wide sequence divergence of satellite DNA could underlie meiotic failure in male hybrids of bighead catfish and North African catfish (*Clarias*, Clariidae). Genomics 116 (4), 110868. 10.1016/j.ygeno.2024.110868 38795738

[B32] Lu M. Li Z. Zhu Z. Y. Peng F. Wang Y. Li X. Y. (2022). Changes in ploidy drive reproduction transition and genomic diversity in a polyploid fish complex. Mol. Biol. Evol. 39 (9), msac188. 10.1093/molbev/msac188 36056821 PMC9486886

[B33] Lukhtanov V. A. Dincă V. Friberg M. Vila R. Wiklund C. (2020). Incomplete sterility of chromosomal hybrids: implications for karyotype evolution and homoploid hybrid speciation. Front. Genet. 11, 583827. 10.3389/fgene.2020.583827 33193715 PMC7594530

[B34] Lutes A. A. Neaves W. B. Baumann D. P. Wiegraebe W. Baumann P. (2010). Sister chromosome pairing maintains heterozygosity in parthenogenetic lizards. Nature 464 (7286), 283–286. 10.1038/nature08818 20173738 PMC2840635

[B35] Macgregor H. C. Uzzell T. M. Jr (1964). Gynogenesis in salamanders related to *Ambystoma jeffersonianum* . Science 143 (3610), 1043–1045. 10.1126/science.143.3610.1043 14112709

[B36] Majtánová Z. Dedukh D. Choleva L. Adams M. Ráb P. Unmack P. J. (2021). Uniparental genome elimination in Australian carp gudgeons. Genome Biol. Evol. 13 (6), evab030. 10.1093/gbe/evab030 33591327 PMC8245195

[B37] Maneechot N. Yano C. F. Bertollo L. A. C. Getlekha N. Molina W. F. Ditcharoen S. (2016). Genomic organization of repetitive DNAs highlights chromosomal evolution in the genus *Clarias* (Clariidae, Siluriformes). Mol. Cytogenet. 9, 4–10. 10.1186/s13039-016-0215-2 26793275 PMC4719708

[B38] Marta A. Tichopád T. Bartoš O. Klíma J. Shah M. A. Bohlen V. Š. (2023). Genetic and karyotype divergence between parents affect clonality and sterility in hybrids. Elife 12, RP88366. 10.7554/eLife.88366 37930936 PMC10627513

[B39] Matute D. R. Butler I. A. Turissini D. A. Coyne J. A. (2010). A test of the snowball theory for the rate of evolution of hybrid incompatibilities. Science 329 (5998), 1518–1521. 10.1126/science.1193440 20847270

[B40] Mogie M. (2013). Premeiotic endomitosis and the costs and benefits of asexual reproduction. Biol. J. Linn. Soc. 109 (2), 487–495. 10.1111/bij.12055

[B41] Monaco P. J. Rasch E. M. Balsano J. S. (1984). “Apomictic reproduction in the Amazon molly, *Poecilia formosa*, and its triploid hybrids,” in Evolutionary genetics of fishes. Editor Turner B. J (New York, NY: Springer), 311–328. 10.1007/978-1-4684-4652-4_6

[B42] Na-Nakorn U. Rangsin W. Boon-ngam J. (2004). Allotriploidy increases sterility in the hybrid between Clarias macrocephalus and *Clarias gariepinus* . Aquaculture 237 (1–4), 73–88. 10.1016/j.aquaculture.2004.02.032

[B43] Neaves W. B. Baumann P. (2011). Unisexual reproduction among vertebrates. Trends Genet. 27 (3), 81–88. 10.1016/j.tig.2010.12.002 21334090

[B44] Newton A. A. Schnittker R. R. Yu Z. Munday S. S. Baumann D. P. Neaves W. B. (2016). Widespread failure to complete meiosis does not impair fecundity in parthenogenetic whiptail lizards. Development 143 (23), 4486–4494. 10.1242/dev.141283 27802173 PMC5201048

[B45] Nguyen D. H. Panthum T. Ponjarat J. Laopichienpong N. Kraichak E. Singchat W. (2021a). An investigation of ZZ/ZW and XX/XY sex determination systems in North African catfish (*Clarias gariepinus*, burchell, 1822). Front. Genet. 11, 562856. 10.3389/fgene.2020.562856 33584785 PMC7874028

[B46] Nguyen D. H. Ponjarat J. Laopichienpong N. Kraichak E. Panthum T. Singchat W. (2021b). Genome-wide SNP analysis suggests male heterogamety in bighead catfish (Clarias macrocephalus,). Aquaculture 543, 737005. 10.1016/j.aquaculture.2021.737005

[B47] Nguyen D. H. Ponjarat J. Laopichienpong N. Panthum T. Singchat W. Ahmad S. F. (2022). Genome-wide SNP analysis of hybrid clariid fish reflects the existence of polygenic sex-determination in the lineage. Front. Genet. 13, 789573. 10.3389/fgene.2022.789573 35186027 PMC8851383

[B48] Ozaki Y. Saito K. Shinya M. Kawasaki T. Sakai N. (2011). Evaluation of *Sycp3* , *Plzf* and *Cyclin B3* expression and suitability as spermatogonia and spermatocyte markers in zebrafish. Gene Expr. Patterns. 11 (5–6), 309–315. 10.1016/j.gep.2011.03.002 21402175

[B49] Patta C. Panthum T. Thatukan C. Wongloet W. Chalermwong P. Wattanadilokchatkun P. (2024). Questioning inbreeding: could outbreeding affect productivity in the North African catfish in Thailand? PLoS One 19 (5), e0302584. 10.1371/journal.pone.0302584 38709757 PMC11073742

[B50] Peters A. H. Plug A. W. Van Vugt M. J. De Boer P. (1997). A drying-down technique for the spreading of mammalian meiocytes from the male and female germline. Chromosome Res. 5 (1), 66–68. 10.1023/a:1018445520117 9088645

[B51] Ponjarat J. Singchat W. Monkheang P. Suntronpong A. Tawichasri P. Sillapaprayoon S. (2019). Evidence of dramatic sterility in F_1_ male hybrid catfish [male *Clarias gariepinus* (Burchell, 1822) × female *C. macrocephalus* (Günther, 1864)] resulting from the failure of homologous chromosome pairing in meiosis I. Aquaculture 505, 84–91. 10.1016/j.aquaculture.2019.02.035

[B52] Pouyaud L. Sudarto Paradis E. (2009). The phylogenetic structure of habitat shift and morphological convergence in Asian *Clarias* (Teleostei, Siluriformes: Clariidae). J. Zool. Syst. Evol. Res. 47 (4), 344–356. 10.1111/j.1439-0469.2008.00507.x

[B53] Qu W. Liu C. Xu Y. T. Xu Y. M. Luo M. C. (2021). The formation and repair of DNA double-strand breaks in mammalian meiosis. Asian J. Androl. 23 (6), 572–579. 10.4103/aja202191 34708719 PMC8577251

[B54] Saito K. Sakai C. Kawasaki T. Sakai N. (2014). Telomere distribution pattern and synapsis initiation during spermatogenesis in zebrafish. Dev. Dynam. 243 (11), 1448–1456. 10.1002/dvdy.24166 25044979

[B55] Schön I. Martens K. van Dijk P. (2009). Lost sex. The evolutionary biology of parthenogenesis (New York: Springer).

[B56] Shimizu Y. Shibata N. Sakaizumi M. Yamashita M. (2000). Production of diploid eggs through premeiotic endomitosis in the hybrid medaka between *Oryzias latipes* and *O. curvinotus* . Zool. Sci. 17, 951–958. 10.2108/zsj.17.951

[B57] Spangenberg V. Arakelyan M. Galoyan E. Matveevsky S. Petrosyan R. Bogdanov Y. (2017). Reticulate evolution of the rock lizards: meiotic chromosome dynamics and spermatogenesis in diploid and triploid males of the genus *Darevskia* . Genes 8 (6), 149. 10.3390/genes8060149 28538689 PMC5485513

[B58] Spangenberg V. Arakelyan M. Simanovsky S. Dombrovskaya Y. Khachatryan E. Kolomiets O. (2024). Tendency towards clonality: deviations of meiosis in parthenogenetic Caucasian rock lizards. Research Square. 10.21203/rs.3.rs-3936576/v2 PMC12358248 40244056

[B59] Stenberg P. Saura A. (2009). “Cytology of asexual animals,” in Lost sex: the evolutionary biology of parthenogenesis. Editors Schön I. Martens K. van Dijk P. (New York: Springer), 63–74.

[B60] Stöck M. Dedukh D. Reifová R. Lamatsch D. K. Starostová Z. Janko K. (2021). Sex chromosomes in meiotic, hemiclonal, clonal and polyploid hybrid vertebrates: along the ‘extended speciation continuum. Philos. T. R. Soc. B. 376 (1833), 20200103. 10.1098/rstb.2020.0103 PMC8310718 34304588

[B61] Stöck M. Lamatsch D. K. Steinlein C. Epplen J. T. Grosse W. R. Hock R. (2002). A bisexually reproducing all-triploid vertebrate. Nat. Genet. 30 (3), 325–328. 10.1038/ng839 11836500

[B62] Tichopád T. Franěk R. Doležálková-Kaštánková M. Dedukh D. Marta A. Halačka K. (2022). Clonal gametogenesis is triggered by intrinsic stimuli in the hybrid’s germ cells but is dependent on sex differentiation. Biol. Reprod. 107 (2), 446–457. 10.1093/biolre/ioac074 35416937

[B63] Yamashita M. Jiang J. Onozato H. Nakanishi T. Nagahama Y. (1993). A tripolar spindle formed at meiosis I assures the retention of the original ploidy in the gynogenetic triploid crucian carp, Ginbuna *Carassius auratus langsdorfii* . Dev. Growth Differ. 35 (6), 631–636. 10.1111/j.1440-169X.1993.00631.x 37281836

[B64] Yang Z. A. Li Q. H. Wang Y. F. Gui J. F. (1999). Comparative investigation on spindle behavior and MPF activity changes during oocyte maturation between gynogenetic and amphimictic crucian carp. Cell Res. 9 (2), 145–154. 10.1038/sj.cr.7290012 10418734

[B65] Yoshikawa H. Morishima K. Fujimoto T. Saito T. Kobayashi T. Yamaha E. (2009). Chromosome doubling in early spermatogonia produces diploid spermatozoa in a natural clonal fish. Biol. Reprod. 80 (5), 973–979. 10.1095/biolreprod.108.075150 19144955

[B66] Yoshikawa H. Morishima K. Kusuda S. Yamaha E. Arai K. (2007). Diploid sperm produced by artificially sex-reversed Clone loaches. J. Exp. Zool. Part A Ecol. Genet. Physiol. 307A, 75–83. 10.1002/jez.a.337 17177281

