# Supplementary material for: Meiotic deviations and endoreplication lead to diploid oocytes in female hybrids between bighead catfish (Clarias macrocephalus) and North African catfish (Clarias gariepinus)
Source: Front Cell Dev Biol. 2024 Aug 23;12:1465335. doi: 10.3389/fcell.2024.1465335 (PMC11377317; doi:10.3389/fcell.2024.1465335)
Supplement: Supplementary file 1 [file DataSheet1.docx]

Supplementary Material

Meiotic deviations and endoreplication lead to diploid oocytes in female hybrids between bighead (*Clarias macrocephalus*) and North African (*Clarias* *gariepinus*) catfish

Dmitrij Dedukh^1+^, Artem Lisachov^2,3+^, Thitipong Punthum^2^, Worapong Singchat^2^, Yoichi Matsuda^2^, Yukiko Imai, Karel Janko^1^, Kornsorn Srikulnath^2,6^*

## * Correspondence: kornsorn.s@ku.ac.th

## Supplementary Figures


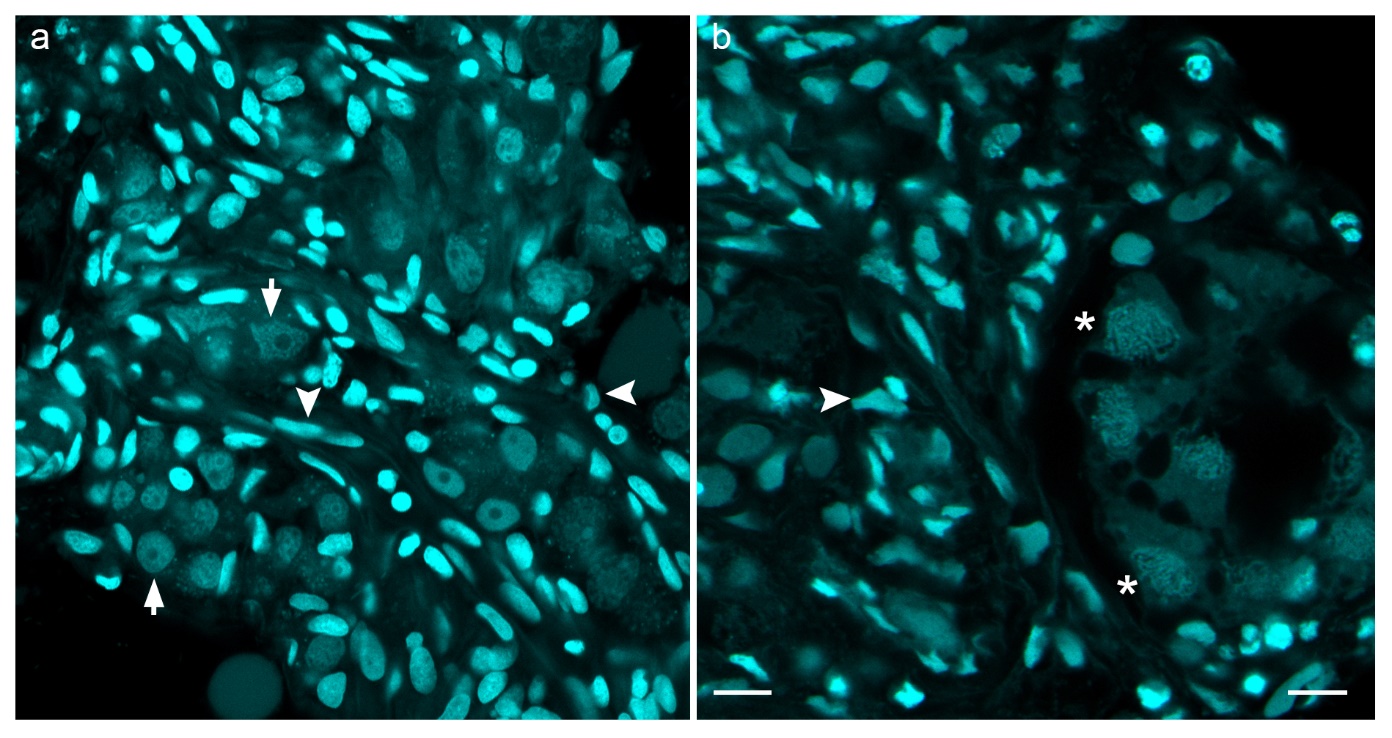


**Supplementary Figure 1.** Microanatomy of ovary from diploid hybrid female with the indication of gonial cells (a), pachytene oocytes (b) and somatic cells (a, b), DAPI staining. Arrows indicate that gonial cells have a greater size and less intensive chromatin staining compared to somatic cells (indicated by arrowheads). Pachytene oocytes are indicated by asterisk. Scale bar represents 10 μm.


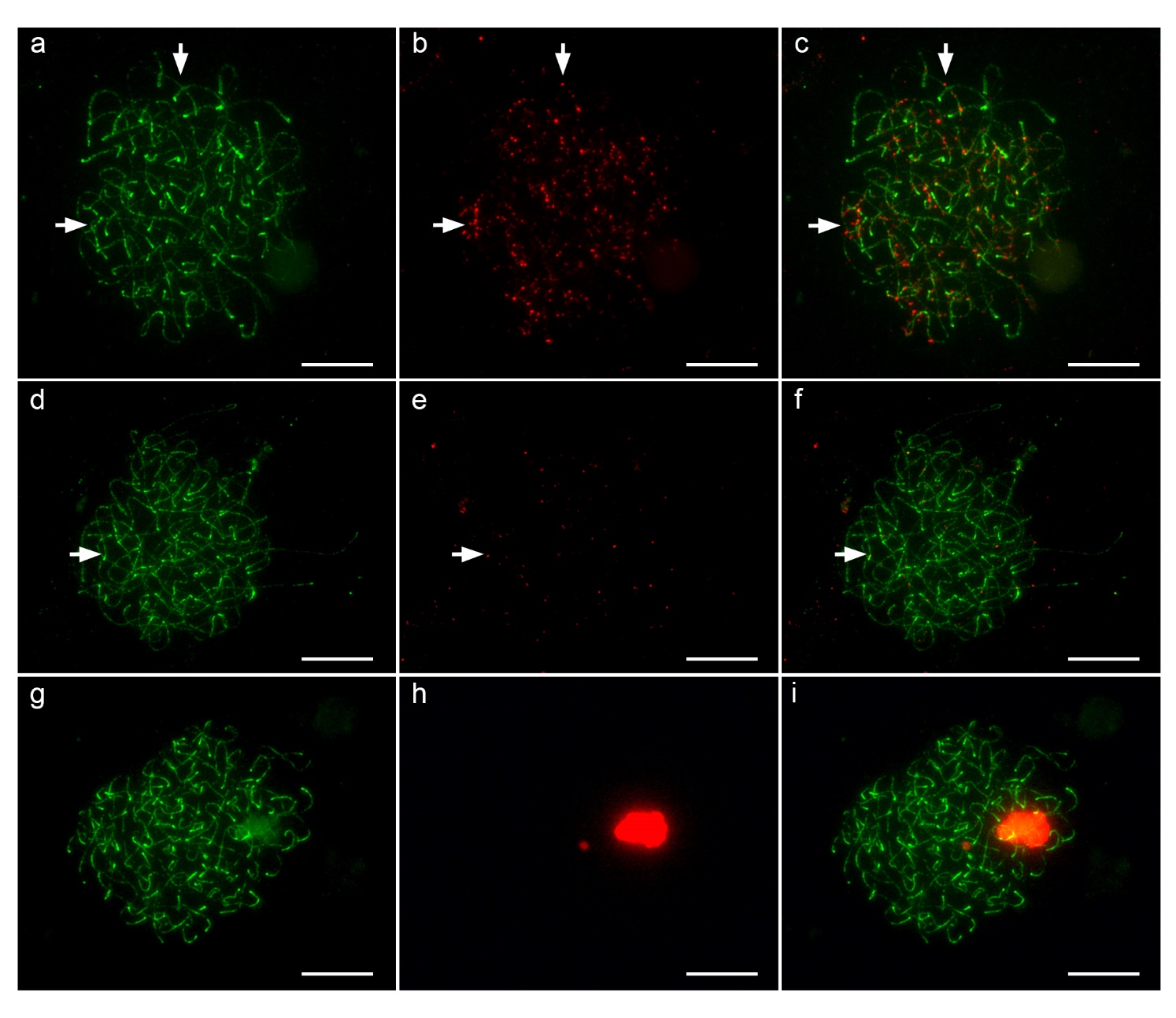


**Supplementary Figure 2.** Immunofluorescent staining of RAD51 foci on synaptonemal complexes from female hybrid catfish. Arrows indicate RAD51 foci (red) with the locations of double-strand breaks in some oocytes (a–c), but almost absent (d–f) or absent (g–i) in most oocytes. Lateral elements of synaptonemal complexes were visualized by SYCP3 staining (green). Scale bars represent 10 μm.


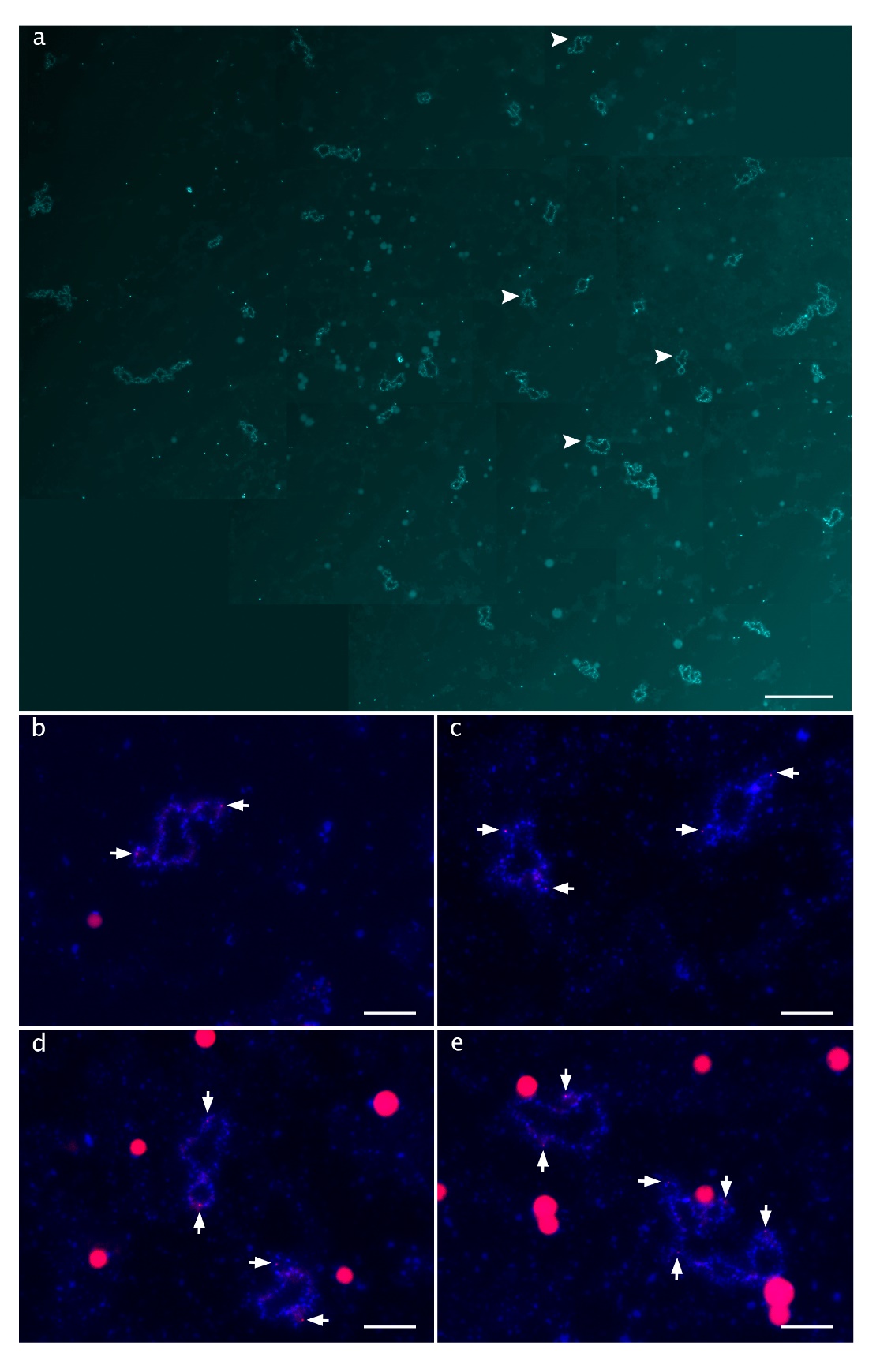


**Supplementary Figure 3.** Chromosomal locations of (TTAGGG)_n_ repeats on bivalents of female hybrid catfish. Full lampbrush chromosomal set that includes DAPI-stained 55 bivalents of female hybrid catfish isolated from individual oocytes (a). Scale bar for (a) indicates 50 μm. Arrowheads indicate enlarged bivalents represented on (b–e). Individual bivalents with (TTAGGG)_n_ repeats detected at the terminal region of the chromosomes (red). Arrows indicate the hybridization signals. Scale bars (b–e) represent 10 μm.


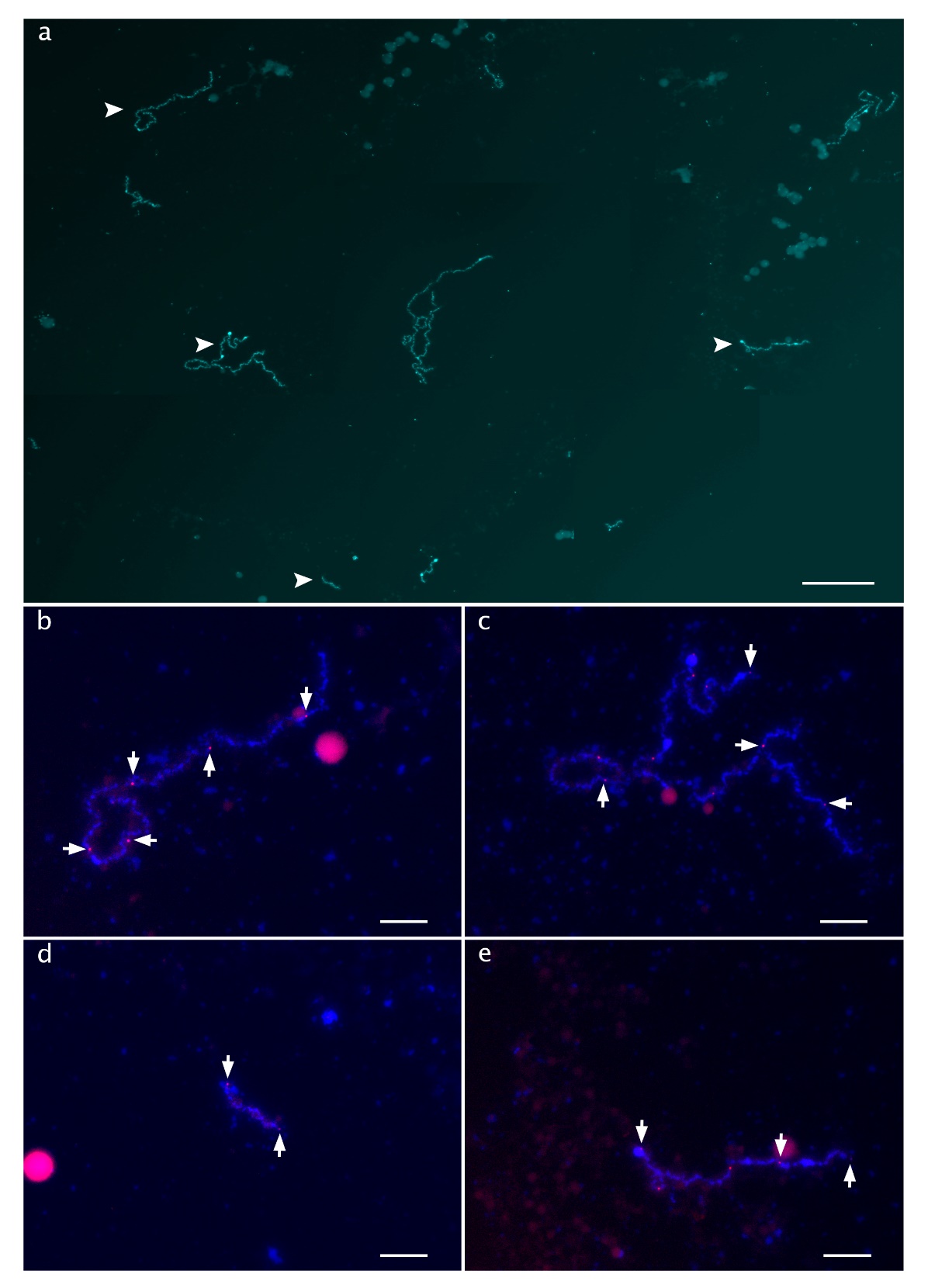


**Supplementary Figure 4.** Chromosomal locations of (TTAGGG)_n_ repeats on univalents of female hybrid catfish. Full lampbrush chromosomal set that includes 55 univalents stained with DAPI (cyan) isolated from individual oocytes (a). Arrowheads indicate enlarged univalents represented on (b–e). Scale bars for (a) represent 50 μm. Individual univalents with (TTAGGG)_n_ repeats detected at the terminal region of the chromosomes (red). Arrows indicate the hybridization signals. Scale bars for (b–e) represent 10 μm.

**
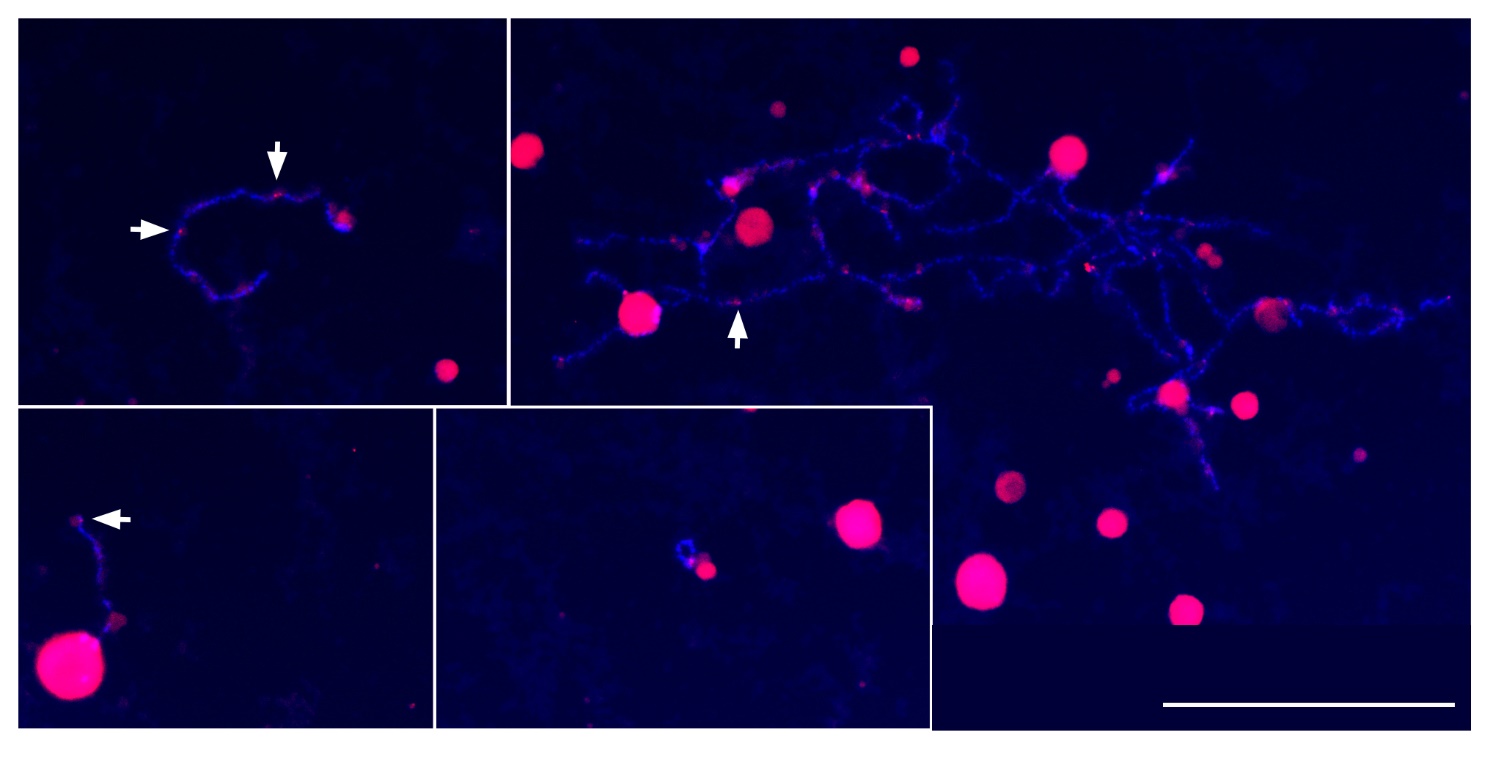
Supplementary Figure 5.** Chromosomal locations of CLA-SAT-225 satDNA pericentromeric sequence (red) on univalents during lampbrush chromosome stage. Full lampbrush chromosomal set that includes DAPI-stained 55 univalents of female hybrid catfish isolated from individual oocytes. Arrows indicate the hybridization signals. Scale bar represents 50 μm.
